# Optimization of UV-Electroproxone procedure for treatment of landfill leachate: the study of energy consumption

**DOI:** 10.1007/s40201-020-00583-9

**Published:** 2021-01-22

**Authors:** Majid Kermani, Abbas Shahsavani, Pegah Ghaderi, Pooria Kasaee, Jamal Mehralipour

**Affiliations:** 1 Research Center for Environmental Health Technology, Iran University of Medical Sciences, Tehran, Iran; 2 Department of Environmental Health Engineering, School of Public Health, University of Medical Sciences, Tehran, Iran; 3 Environmental and Occupational Hazards Control Research Center, Shahid Beheshti University of Medical Sciences, Tehran, Iran; 4 Department of Environmental Health Engineering, School of Public Health, Shahid Beheshti University of Medical Sciences, Tehran, Iran; 5 Master of Environment Engineering Water and Wastewater, West Tehran Branch Islamic Azad University, Tehran, Iran; 6 Master of Civil Engineering, Azad University of Tehran West Branch, Tehran, Iran; 7 Student Research Committee, Iran University of Medical Sciences, Tehran, Iran; 8 Environmental Health Engineering, Iran University of Medical Sciences, Tehran, Iran

**Keywords:** Optimization, UV-Electroproxone process, landfill leachate, Design Expert software

## Abstract

With increased population, treatment of solid waste landfill and its leachate is of major concern. Municipal landfill leachate shows variable, heterogeneous and incontrollable characteristics and contains wide range highly concentrated organic and inorganic compounds, in which hampers the application of a solo method in its treatment. Among different approaches, biological treatment can be used, however it is not effective enough to elimination all refractory organics, containing fulvic-like and humic-like substance. In this experimental study, the UV Electroperoxone process as a hybrid procedure has been employed to treat landfill leachate. The effect of various parameters such as pH, electrical current density, ozone concentration, and reaction time were optimized using central composite design (CCD). In the model fitting, the quadratic model with a P-Value less than 0.5 was suggested (< 0.0001). The R^2^, R^2^ adj, and R^2^ pre were determined equal to 0.98,0.96, and 0.91 respectively. Based on the software prediction, the process can remove 83% of initial COD, in the optimum condition of pH = 5.6, ozone concentration of 29.1 mg/l. min, the current density of 74.7 mA/cm^2^, and process time of 98.6 min. In the optimum condition, 55/33 mM H_2_O_2_ was generated through electrochemical mechanism. A combination of ozonation, photolysis and electrolysis mechanism in this hybrid process increases COD efficiency removal up 29 percent which is higher than the sum of separated mechanisms. Kinetic study also demonstrated that the UV-EPP process follows pseudo-first order kinetics (R^2^ = 0.99). Based on our results, the UV-EPP process can be informed as an operative technique for treatment of old landfills leachates.

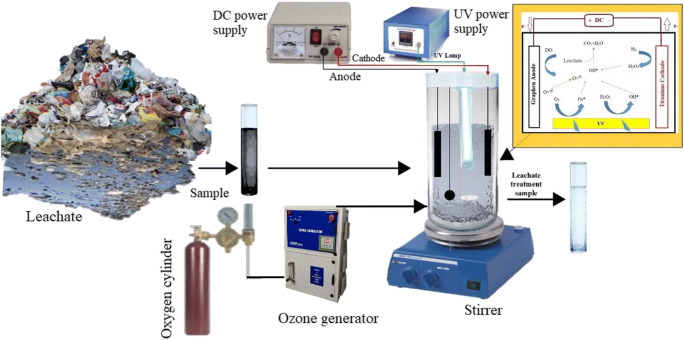

## Introduction

In recent decades, a considerable increment of annual solid waste production (8–10%) has been experienced all over the world [1]. The main drawback imposed on landfilling is leachate generation. The refractory organics, like fulvic-like and humic-like materials, linger in the leachate, especially old leachate. The release of this wastes can cause important environmental concerns due to production of more harmful and toxic compounds in leachate [2]. Therefore, advanced treatment is necessary to destroy these refractory organics and improve biodegradability before discharge [3, 4]. Ozonation has been increasingly used for treating the resistant organic compound [5, 6]. In ozonation, organic contaminants can be oxidized by ozone molecules and/or hydroxyl radicals (OH^●^) produced from the decay of ozone molecules [7, 8]. Throughout ozonation, uncompleted degradation of refractory pollutant occurs. To overcome this dilemma, ozonation has been conservatively applied with other methods like ultraviolet irradiation (UV/O_3_) and H_2_O_2_ (H_2_O_2_/O_3_, i.e., peroxone process) as an advance oxidation process (AOP_s_) [9]. In the peroxone process, hydroxyl radicals were generated (Eqs. 1, 2) [10].

1 $${H}_{2}{O}_{2}+{O}_{3}\to {OH}^{\bullet }+{O}_{2}^{\bullet }+{O}_{2}$$

2 $${O}_{3}+{H}_{2}{O}+e\text{-}\rightarrow{HO}^\bullet+O^\text{-}_2+OH^\text{-}$$

Advance Oxidation Process (AOPs), which depend mainly on the formation of the free radicals to destroy organic materials, have revealed good prospective in the treatment of organic pollutants [1]. In hybrid AOPs (HAOPs), combination of two or more mechanisms are utilized for generation of oxidants such as OH^•^. The advantage of HAOPs is high-performance oxidation and mineralization of resistant organic compounds without the possibility of producing intermediates and secondary waste [2]. One common (HAOP_s_) is the electro-proxone process (EPP) [3]. In the EPP, the required ozone is produced through an ozone generator, while hydrogen peroxide is formed in the surface of cathode [3]. Further, the electrically produced H_2_O_2_ reacts with ozone and consequently, OH^•^ is formed (Eq. 1) [1]. With the purpose of accelerating the production of OH^•^, ultraviolet (UV) irradiation can be employed in (EPP) as the photoelectro-peroxone process (UV-EPP or Photo- EPP) [11]. According to Eqs. 3–5, Photolysis of ozone and H_2_O_2_ produces OH^●^ [3, 12]. The benefits of utilizing UV-EPP method are its manageable system, controlled production of hydrogen peroxide without the need of adding excess amount, no sludge producing and its simple, environmentally friendly process [1, 5].

3 $${H}_{2}{O}_{2}+UV\to 2{HO}^{\bullet }$$

4 $${O}_{3}+{H}_{2}O+UV\to 2{HO}^{\bullet }+{O}_{2}$$

5 $${O}_{3}+UV\to O+{O}_{2}$$

Response surface methodology (RSM) is the combination of mathematical and statistical methods to investigate the impact of different variables of one process [13–15]. Previous reports have been studied the applicability of different AOPs as per or post-treatment of leachate. for instance, Wang and et al. [16] used the E^+^-ozonation technique for concentrated leachate disposal. Ma and et al.[17] used catalytic micro-ozonation through Fe_3_O_4_ nanoparticles @ cow-dung ash for advanced treatment of biologically pre-treated leachate. In this research, the effect of important operating factors on UV-EPP (i.e., pH, current density, the concentration of initial ozone, and reaction time) were studied via central composite design (CCD). In addition, the consumed electrical energy, Kinetic of reactions and the synergist effect were investigated in optimum condition.

Finally, we applied novel AOPs to the treatment of leachate. This process is a hybrid process that photolysis, electrolysis, and simple ozonation mechanisms are used to direct and indirect oxidation of organic matter in leachate. The goal of UV-EPP was the generation of free oxidation radicals, and enhance the efficiency.

## Materials and methods

### Landfill leachate characteristics

**Fig. 1:**
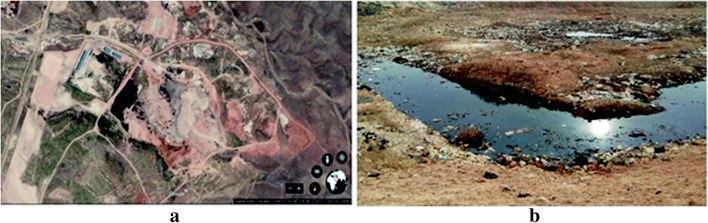
**a** Satellite image, **b** the real image of the landfill site

The untreated landfill leachate was collected from the Hamadan sanitary landfill location placed in Hamadan province, west of Iran (34● 57’52 N and 48● 37’08 E) (Fig. 1). Landfill leachate was collected in 4 L glass vessels and kept in a refrigerator at 4–6 °C. the main characteristics were measured according to the instructions given in the reference of water and wastewater examination [18]. Some of main characteristics of the landfill leachate have been presented in Table 1. The satellite and real image of the leachate accumulation in the Hamadan landfill site have been shown in Fig. 1.

**Table 1:** Main characteristics of the landfill leachate

Constituent	pH	Ammonia nitrogen (mg/L)	COD (mg/L)	BOD_5_ (mg/L)	TOC (mg/L)	BOD_5_/COD ratios	TP (mg/L)	TDS (mg/L)
Value	8 ± 0.8	2178 ± 98	9433 ± 348	1500.7 ± 214.9	6647 ± 243.6	0/15	120	1200

### Chemical and reagents

Sulfuric acid (H_2_SO_4_ 96%) and sodium hydroxide (NaOH 98%) were purchased from Merck Company, COD vials (high range 0-1500 mg/l) were prepared from Laviband Company. The analysis was performed by spectrophotometer (DR6000 HACH Co.), Low pressure mercury lamp (254 nm, 6 W, Philips Morgian, USA (, Oxygen and Ozone generator (PORSA model, ARDA Co. France), pH meter (HACH Co.), DC source (5.00 A, 50 V, P405, ADAK Co.), Graphene electrodes (20 cm × 2 cm, Etemad Gostar Iranian Co.), and impugner (1 liter, DURAN Co.).

### Experimental procedures

**Fig. 2:**
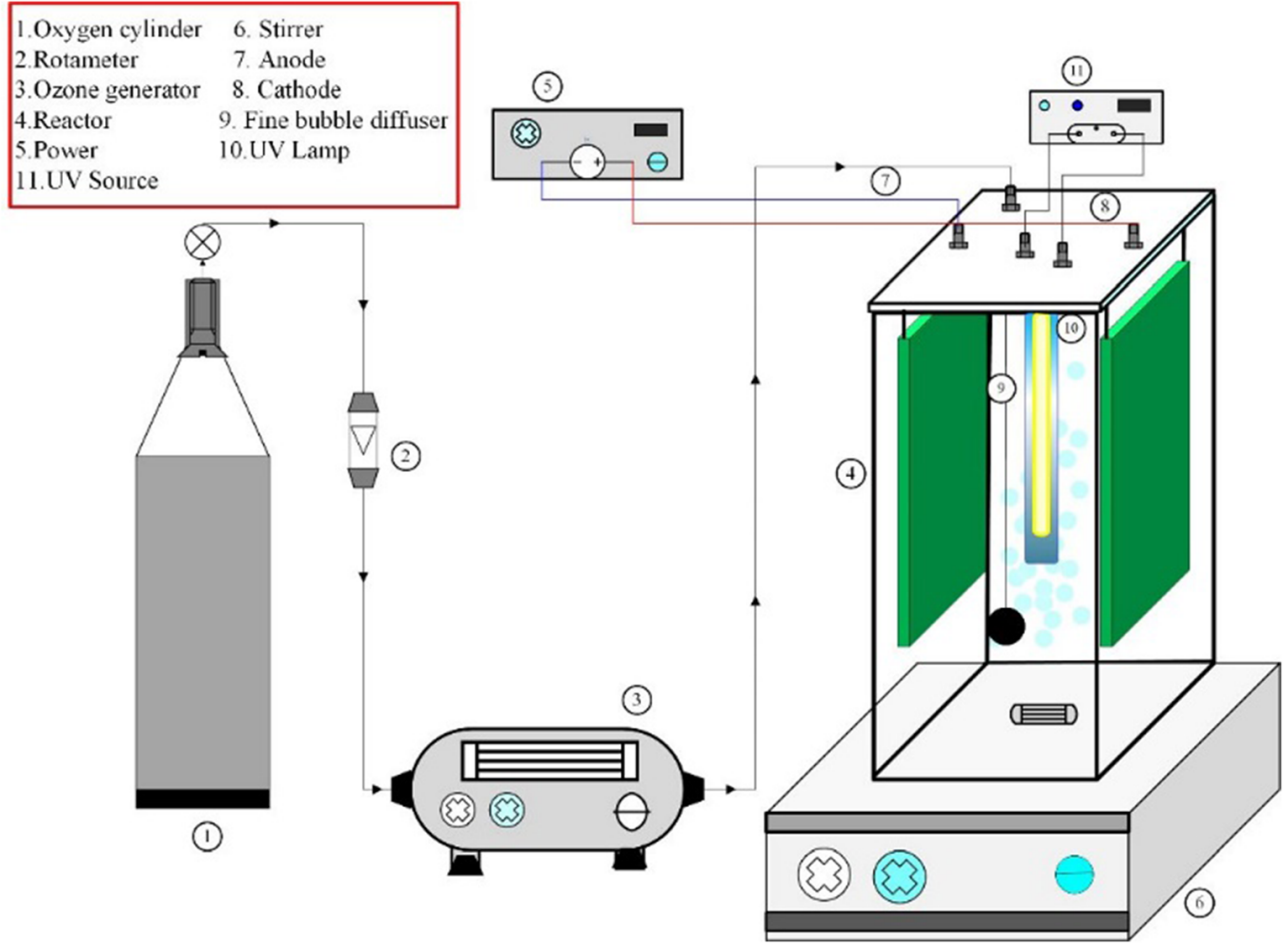
Schematic view of UV-EPP reactor

UV-EPP pilot consists of a one-liter batch quartz cylinder covered by UPVC box (Fig. 2). The experiments were conducted at room temperature (25 ± 3). Two pairs of graphene electrodes as cathodes and anodes immersed in the middle of the reactor were used. The distance between the electrodes was 1.0 cm. Ozone gas was constantly produced from a pure O_2_ feed gas (99.9%) through an ozone generator and then diffuse into the aqueous by fine bubble diffuser. The ozone concentration in the gas (O_2_/O_3_ mixture) can be tuned via altering the power of ozone generator. The Direct electric current (DC) power supply was applied to employ electrical current in controlled conditions. For UV irradiation, a low-pressure UV-C lamp (254 nm, 16 W) emission wavelength in the quartz tube was used such that the lamp was located axially inside the reactor. In this condition, UV intensity was 2.3 mW/cm^2^ (corresponding to a photon fluency rate of 1.84 × 105 Einstein m^2^. s^− 1^). The content of the reactor was mixed with a magnetic stirrer (120 rpm). Each mechanism (electrolysis, UV-photolysis, ozonation) was carried out at the same conditions and their synergist effect was also investigated, separately. In each run, 1000 mL of leachate was entered into the reactor. The initial pH of the leachate was adjusted with H_2_SO_4_ and NaOH (0.1 N). The initial and final concentrations of COD were measured according to the instructions given by the standard method [1]. The UV-EPP was initiated by concurrently employing a continuous current to electro-generate H_2_O_2_, UV irradiation, and sparging ozone-containing oxygen gas over the reactor. After each step of the run, 5 minutes of nitrogen stripping was applied to eliminate the remaining ozone gas in the solutions. The efficiency of COD was calculated by the following equation (Eq. 6).

6 $$COD removal\%=\frac{Cf-C0}{C0} \times 100$$

Where C_0_ and C_f_ refer to the COD concentrations in the sample of leachate before and after the reaction, respectively.

### Electrical energy consumption calculations

In the optimum condition of UV-EPP, the specific energy requirements (SER) for COD elimination throughout the ozonation, electrolysis, photolysis, and UV-EPP are survey via (Eqs. (7–10)), separately. Ozone consumption (CO_3_) in ozonation and UV-EPP was calculated according to Eq. (11). The normal energy needed for ozone formation was expected to be 10 kWh kg^− 1^ O_3_ [19].

7 $$\text{S}\text{E}\text{R} \text{e}\text{l}\text{e}\text{c}\text{t}\text{r}\text{o}\text{l}\text{y}\text{s}\text{i}\text{s}(\text{k}\text{W}\text{h}/\text{g} \text{C}\text{O}\text{D} \text{r}\text{e}\text{m}\text{o}\text{v}\text{e}\text{d}) =\frac{UIT}{\left(\text{C}\text{O}\text{D}0-\text{C}\text{O}\text{D}\text{t}\right)\text{V}}$$

8 $$\text{S}\text{E}\text{R} \text{p}\text{h}\text{o}\text{t}\text{o}\text{l}\text{y}\text{s}\text{i}\text{s}(\text{k}\text{W}\text{h}/\text{g} \text{C}\text{O}\text{D} \text{r}\text{e}\text{m}\text{o}\text{v}\text{e}\text{d}) =\frac{Wt}{\left(\text{C}\text{O}\text{D}0-\text{C}\text{O}\text{D}\text{t}\right)\text{V}}$$

9 $$\text{S}\text{E}\text{R} \text{o}\text{z}\text{o}\text{n}\text{a}\text{t}\text{i}\text{o}\text{n}(\text{k}\text{W}\text{h}/\text{g} \text{C}\text{O}\text{D} \text{r}\text{e}\text{m}\text{o}\text{v}\text{e}\text{d}) =\frac{rCO3}{\left(\text{C}\text{O}\text{D}0-\text{C}\text{O}\text{D}\text{t}\right)\text{V}}$$

10 $$\text{S}\text{E}\text{R} \text{U}\text{V}-\text{E}\text{P}\text{P}(\text{k}\text{W}\text{h}/\text{g} \text{C}\text{O}\text{D} \text{r}\text{e}\text{m}\text{o}\text{v}\text{e}\text{d}) =\frac{UIT\times Uphotolysis\times rCO3}{\left(\text{C}\text{O}\text{D}0-\text{C}\text{O}\text{D}\text{t}\right)\text{V}}$$

11 $$\text{C}\text{O}3 =\text{Q}\text{g}?\left(\left[\text{O}3\right]\text{i}\text{n}\text{l}\text{e}\text{t}-\left[\text{O}3\right]\text{o}\text{u}\text{t}\text{l}\text{e}\text{t}\right)\text{d}\text{t}$$

SER_Electrolysis_, SER_photolysis_ SER_ozonation_, and SER_UV−EPP_ are the specific energy requirement (kWh/g COD removed) for electrolysis, photolysis, ozonation, and UV-EPP, respectively; U is the cell voltage (V), I is the current (A), T is the reaction time (h), r is the energy requirement for ozone production (10 kWh/kg ozone), The minimal power of the UV lamp is W (10 W) and corrected considering 60% lamp effectiveness consistent with the manufacturer’s manual, V is the solution volume (L), CO_3_ is the total ozone spent in ozonation and UV-EPP (g), Qg is the gas flow rate (L/min), [O_3_] inlet and [O_3_] outlet are the concentration of gas phase ozone (mg/L) at the reactor gas inlet and outlet, respectively [20].

### Analytical methods

The concentrations of ozone at the reactor’s inlet and outlet were constantly checked by ozone analyzer (UV-300, Simson EP Hi-Tech Co.) in UV-EPP and ozonation process. The concentration of ozone in the aqueous was detected via the indigo technique [2]. The COD of leachate was measured via DR6000 spectrophotometer. H_2_O_2_ and OH^●^ was calculated by the Terephthalate method [21].

### Experimental design

The design of the experiment (DOE) typically is used to optimize the effective variables in the UV-EPP process to increase characteristics performance and decrease the experiments’ error [22]. Here in, for the design, analysis, modeling, and predicate of the optimum condition in COD removal, the central composite design (CCD) via Design Expert Software (version 11) was used. The CCD is a mixture of mathematical and statistical procedure that was used to identify major variables and optimize the conditions. This method is an advanced technique of factorial design that provides precise models for curvature via considering the relations of factors. The level and range of factors were studied at five levels (Table 2). According to the CCD design, 30 experiments were planned (Table 4). The calculated responses involved the COD removal in different runs in UV-EPP. The CCD based results were investigated via ANOVA. The multi-degree coefficients of Eq. 12 were applied for determining the coefficients [23]. P-value with a 95% confidence level was employed to estimate the model functions effect.

12 $$Y={\beta }_{0}+{\beta }_{i}{X}_{i}+{\beta }_{j}{X}_{j}+{\beta }_{ii}{X}_{i}^{2}+{\beta }_{jj}{X}_{j}^{2}+{\beta }_{ij}{X}_{i}{X}_{j}$$

Y, i, j, b, X are process response, linear coefficient, quadratic coefficient, regression coefficient and coded independent variables, respectively. β is the correlation coefficient.

**Table 2:** Summary of design of UV-EPP based on CCD

symbol	name	unit	Min level	Max level	Low level	High level	mean	Sd.
A	pH	-	2	10	4	8	6	1.789
B	O_3_ concentration	mg/l.min	10	50	20	40	30	8.944
C	Direct Current(DC)	mA/cm^2^	18.75	93.75	56.25	75	65/25	16.771
D	Reaction Time(RT)	min	25	125	50	100	75	22.361

Then the single term impact and the interactions between the variables (pH, ozone concentration, direct current and reaction time) on the UV-EPP process efficiency fitted and optimized via the quadratic polynomial model. This model simulates the process efficiency in the face of five linear variables, ten interacting factors, and five curved variables. The correlation coefficients (R^2^, R^2^ adj. R^2^ predict) help to deduce the interactions of factors graphically and find the best performance in different conditions based on the surface of the three-dimensional response plots.

## Results and discussion

### Design of experiments

Initially, lack of fit teste (Table 3) for Linear, 2FI, quadratic and cubic models was done and this model was chosen for other analysis steps, due to the minor lack of fit and maximizing the attuned R-squared and the anticipated R-squared of the quadratic model.

**Table 3:** Lack of fit teste for CCD analysis

Source	Sum of squared	Degree of freedom	Mean squares	F-value	*p*-value Prob > F	F-value	*p*-value Prob > F	
						Lack of fit		
Linear	352.5	4	88.13	13.14	< 0.0001	14.54	0.0038	
2FI	408.25	10	40.83	6.93	0.0002	13.75	0.0049	
Quadratic	510.25	14	36.45	55.13	< 0.0001	8.25	0.4258	Suggested
Cubic	515.75	22	23.44	37.16	< 0.0001	1.14	0.3296	

The statistical model was used to find the optimal approximation of the system response. In the CCD, the experiments were planned in a random activity to minimize the effect of uncontrolled variables and errors. As presented (Tables 2 and 4), four independent variables (pH, ozone concentration, direct current, and reaction time), were selected in five levels as coded value (-a, 1, 0, + 1, +a) and the responses of all observed 30 experiments was recovered and presented. Based on Table 4, the minimum and maximum efficiency of UV-EPP in the removal of COD was 63 and 79 percent, respectively. Analysis of variance was also performed to find the most significant variables effect and the interactions between them. The results were analyzed by ANOVA at 95% confidence level to fit the experimental results (Table 5). The P-Value and F-Value in the ANOVA were used to determine the role of each variable. The low P-Value (less than 0.05), indicated the significance of the selected model. In this model, P-Value above 0.05, which reduced the statistical effect of significant variables, were removed and statistically significant related variables and interactions were included. Also, the lack of fit P-value confirms the significance and usability of the model.

**Table 4:** Designed experiments based on CCD

Run	pH	Ozone(mg/l.min)	DC(A)	Reaction time(min)	Observed COD removal (%)	Predicted COD removal (%)
1	6	30	56.2	125	78	78.3
2	4.00	20.00	75.00	100.00	79	78.95
3	6.00	30.00	56.25	25.00	67	67.3
4	6.00	30.00	93.75	75.00	73	73.3
5	6.00	30.00	56.25	75.00	75	74.8
6	10.00	30.00	56.25	75.00	71	71.5
7	4.00	20.00	37.50	100.00	71	70.7
8	6.00	10.00	56.25	75.00	69	69.3
9	8.00	40.00	37.50	100.00	70	69.7
10	4.00	40.00	37.50	100.00	70	69.95
11	8.00	40.00	75.00	50.00	71	70.7
12	6.00	30.00	56.25	75.00	75	74.8
13	8.00	20.00	37.50	100.00	72	67.95
14	6.00	30.00	56.25	75.00	75	74.8
15	4.00	40.00	75.00	100.00	78	77.7
16	6.00	50.00	56.25	75.00	70	70.3
17	8.00	20.00	75.00	50.00	65	64.95
18	6.00	30.00	18.75	75.00	63	63.3
19	8.00	20.00	75.00	100.00	78	77.7
20	8.00	20.00	37.50	50.00	65	64.7
21	4.00	20.00	75.00	50.00	69	68.7
22	6.00	30.00	56.25	75.00	74	77.8
23	8.00	40.00	75.00	100.00	76	75.95
24	4.00	20.00	37.50	50.00	67	66.95
25	6.00	30.00	56.25	75.00	76	74.8
26	4.00	40.00	75.00	50.00	72	71.95
27	6.00	30.00	56.25	75.00	74	74.83
28	2.00	30.00	56.25	75.00	75	75.3
29	8.00	40.00	37.50	50.00	68	6.95
30	4.00	40.00	37.50	50.00	69	68.7

**Table 5:** ANOVA of the fitted models for COD removal in UV-EPP

Source	Sum of Squares	df	Mean Square	F- Value	*p*-value Prob > F	
Model	510.25	14	36.45	55.13	< 0.0001	significant
A-pH	13.50	1	13.50	20.42	0.0004	
B-Ozone concentration	4.17	1	4.17	6.30	0.0240	
C-Applied current	130.67	1	130.67	197.65	< 0.0001	
D-Reaction time	204.17	1	204.17	308.82	< 0.0001	
AB	0.25	1	0.25	0.38	0.0478	
BC	1.00	1	1.00	1.51	0.0277	
BD	25.00	1	25.00	37.82	< 0.0001	
CD	25.00	1	25.00	37.82	< 0.0001	
A^2	3.86	1	3.86	5.83	0.0289	
B^2	42.86	1	42.86	64.83	< 0.0001	
C^2	72.43	1	72.43	109.56	< 0.0001	
D^2	6.86	1	6.86	10.37	0.0057	
Residual	9.92	15	0.66			
Lack of Fit	7.08	10	0.71	1.25	0.4258	Not significant
Pure Error	2.83	5	0.57			
Cor Total	520.17	29				

The P-Value is less than 0.05 for the variables and their interactions, which means their statistical influence on the process. Based on the analyzed results, F-Value and P-value models were determined (< 0.0001) and 55.13, respectively. The lack of fitted P-value(0.4258) was more than 0.05, so, indicating the considerably fitted model. The fitting regression model was also used to determine the effect of variables (Eq. 13).

Efficiency(%) = 29.7+(0.5 × pH)+(1.0 × Ozone)+(0.4 × Current(+(0.2 × Time)+(0.006 × pH × Ozone)+(0.001 × Ozone × Current)–(0.005 × Ozone × Time)+(0.002 × Current × Time)+(0.1 × pH2)-(0.01 × Ozone2)-(0.003 × Current2)-(0.0008 × time2 ) [13].

R^2^, R^2^ Adj and R^2^ predict (0.98, 0.96 and 0.91, respectively) indicated the great relationship between experimental and predicted values.

#### Effect of parameters

**Fig. 3:**
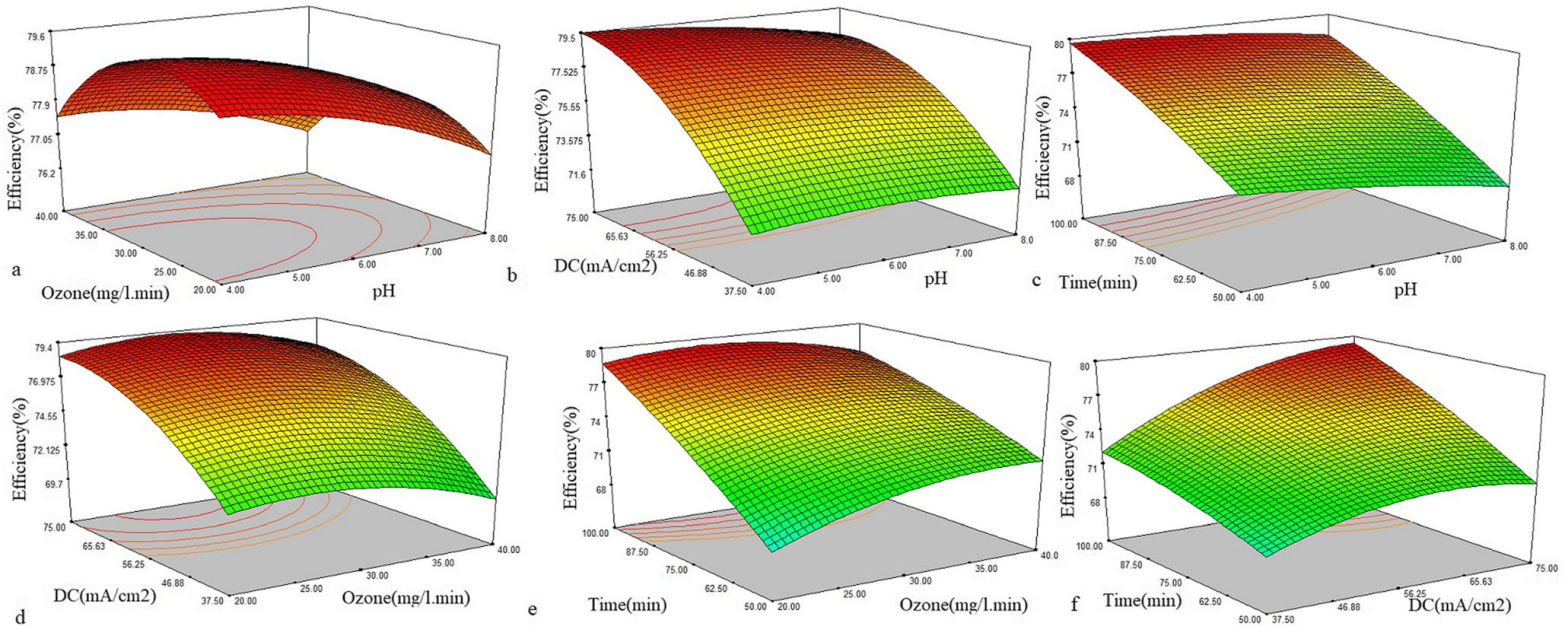
Response surface plots

Here in, response surface plots are presented to determine the single-terms and effect of interactions between variables in the removal of COD in UV-EEP. The three-dimension surface response provides important information about the interactions between the variables (Fig. 3).

Figure 3 demonstrates the variation of the COD removal efficiency as a function of the initial pH and ozone concentration, while the other variables were considered constant in the central point values (DC = 65/25 mA/cm2, RT = 75 min). The efficiency of UV-EPP was increased with increasing ozone gas concentration up to a certain point (middle concentration) and then the efficiency was decreased. The COD removal efficiency was decreased by increasing the pH which is an effective parameter on the chemical processes, especially AOPs. This parameter as a, directly and indirectly, influence on the processes. In AOPs, changing the pH value, affect the rate of radicals’ production [24]. The results indicated that the efficacy of the process for COD removal was closed in the selected range of pH value. However, with increasing the pH value from acidic to alkaline, efficiency was relatively reduced. According to previous studies, in the conventional ozonation process, through increasing the pH to the alkaline range, the ozone molecule reacts with the hydroxyl ion (OH^−^) and produces HO_2_¯ ion (Eq. 13). Further, this ion reacts with the ozone molecule and generates OH^●^ (Eq. 14).

13 $${O}_{3}+{OH}^{-}?{HO}_{2}^{-}+{O}_{2}$$

14 $${HO}_{2}^{-}+{O}_{3}\to {OH}^{\bullet }+{O}_{2}^{\bullet -}+{O}_{2}$$

In the UV-EPP, an affirmative condition for H_2_O_2_ electro-generation is generated in a slightly acidic solution. While, in alkaline solution, H_2_O_2_ only formed via electro generation and produce oxygen and H_2_O molecule through self-destruction. Furthermore, in the electrochemical production of H_2_O_2_/HO_2_, the formation of HO_2_^−^ is a sub-reaction between the hydroxyl ion and the ozone molecule, which lead to reduction of the ozone in the solution. This sub-reaction decreases reaction rate of ozone with H_2_O_2_ and HO_2_, resulting in less creation of radical hydroxylation. On the other hand, due to the reduction of ozone and increasing the HO_2_^−^ amount relative to ozone, HO_2_^−^ acts as a radical scavenger and competes with the pollutant in radical hydroxyl consumption (Eq. 15).

15 $${HO}_{2}^{-}+{OH}^{\bullet }\to {H}_{2}{O}^{\bullet }+{OH}^{-}$$

The most important point of this process is the favorable efficiency of in a wide range of pH value. Since the industrial wastewater and leachate have a varied pH, so UV-EPP can be used as a promising method to treat the pollutants. In previous studies, several researchers reported the effect of pH value on the efficiency of Electroproxone processes. Yujue Wang et al. [10] reported the EP process with similar result in 3 and 7 pH values in the degradation of Orange II dye. Gang Yu et al. [25] also reported the similar result of EP process in 3 and 7 pH values in the degradation of ibuprofen. furthur in pH equal to 10, the efficiency of EP process was decreased.

According to the mass transfer theory, enhancing the amount of ozone intake into the reactor increases the concentration of soluble ozone in the aqueous. The ozone gas has a direct and indirect role in UV-EPP, i: direct oxidation of organic pollutant via ozone gas, ii: decomposition of ozone molecule and reaction with H_2_O_2_ to produce OH^●^ as a non-selective oxidant. The efficiency of the process can be developed by increasing the ozone input to the reactor up to a certain concentration of ozone molecules. However, due to the weak solubility of ozone in solution, transfering from the gas phase to the liquid phase is limited. In other words, increasing inlet concentration of ozone does not lead to more immigration of ozone gas to the aqueous phase [26]. The Wang et al. [27] results indicated that the efficiency is improved via increasing ozone concentration in MB removal by EP and the highest ozone concentration shows highest efficiency. The report by Zhaoxin Li et al. [28] showed that via increasing ozone concentration in the degradation of refractory organic pollutants in landfill leachate, the efficiency of the process was improved and high performance earns in 154 mg/l O_3_. In Kermani et al. [29] study reported that the high efficiency of EPP was obtained at slightly acidic pH.

According to Fig. 3b, the pH value and the applied direct current were independent variables and other factors were considered constant in the central point values (O_3_ = 30 mg/l.min, RT = 75 min). Based on results, the direct current parameter has a significant effect on UV-EPP efficiency and its high value lead to improved efficiency in COD removal. This is due to the fact that increasing applied electrical current enhances the electro-production of H_2_O_2_ at the graphene-based cathode. As a result, hydroxyl radicals are produced more than the amount produces in the reaction between ozone and H_2_O_2_. Furthermore, high applied current causes more cathodic activation and anodic direct oxidation of ozone molecules. However, after the optimum value of applied current, the efficiency of the process became stable. For this subject, two reasons are involved: (i) the ability to react between H_2_O_2_ and ozone molecules is limited because certain amount of ozone gas dissolves in the solution. (ii) through increasing the applied current, interfaces reaction was accrued in solution, in which caused disturbing the H_2_O_2_ electro-generation and the production of a water molecule happened via oxygen reduction. Also, higher current of H_2_O_2_ was oxidized in anode part (Eq. 16).

16 $${H}_{2}{O}_{2}?{O}_{2}+2{H}^{+}+2{e}^{-}$$

In other hands, the low dissolution of ozone molecules in the solution caused a dramatic decreasing in the rate of H_2_O_2_ converting to the OH^●^ and H_2_O_2_ remain in the solution. Since H_2_O_2_ is not a powerful oxidant, the process efficiency is reduced [4]. Qiu et al. [30] investigated the p-nitro phenol mineralization through a combined process. The result indicated that an optimum electrical current for the removal of pollutants is 100 mA. In report by Ahmadi et al. [31], photoelectro-peroxone/ZVI process was used for the degradation of organic pollutants. The result showed that 300 mA applied current has the highest efficiency among other values (0,100,300 and 400 mA applied current). Also in Bernal-Martinez at al [32], examined the effect of synergy of EC and ozonation processes in industrial wastewater treatment. In the given study, 10–40 mA cm^− 2^ current density and 5 mg L^− 1^ ozone concentration were applied. The maximum of COD removal efficiency (84%) was achieved in current densities of 20 mA cm^− 2^ and pH of 7.

According to Fig. 3c, pH value and reaction time were independent parameters and other variables were considered constant in the central point values (DC = 65/25 mA/cm^2^, O_3_ = 30 mg/l.min). Based on results, the reaction time as a variable has a significant effect on UV-EPP efficiency and longer reaction time caused improved COD removal. Also pH has the same effect in the selected range as shown in Fig. 3a. Obviously, by increasing the concentration of pollutants in the solution, more oxidants such as OH^●^, and Ozone molecules are consumed. Therefore, higher reaction time is necessary for mineralization because other parameters such as photolysis, electrolysis, ozonation and OH^●^ are instant values. In Mina Gharchi et al. [33], repoted that significat removal of COD obtained in 3 h in optimum condition.

After optimizing the process and determining the proposed optimal conditions by the model, pH = 5.62, ozone concentration = 29.11 mg/l.min, current density = 74.71 mA/cm^2^, and reaction time = 98.63 was proposed by software. According to the predicate point, the efficiency of COD removal in optimum condition was 84.83 and 83 percent as a theatrical and experimental removal via UV-EPP.

### H_2_O_2_, and OH^●^ production during optimum condition

**Fig. 4:**
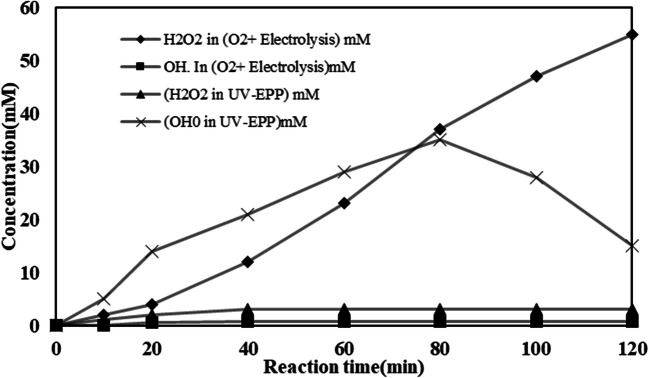
H_2_O_2_ and OH^●^ production in optimum condition

Through using graphene as a cathode and sparged O_2_ gas to the reactor, the H_2_O_2_ concentration increased linearly with electrolysis time and reached to 1.95 gr (55/33 mM) under 74.71 mA/cm^2^ of current density after 120 min electrolysis (Fig. 4).

These results indicated that the ozone quickly reacted in situ with the electrogenerated H_2_O_2_ to produce OH^●^(Fig. 4), that was successively taken by TA to generate HTA. Moreover, owing to the particularly OH^●^ short life, this radical cannot collect in a solution. On the other hand, the continuous increase of HTA concentration as electrolysis point out the constant generation of OH^●^ in this system. Bo Yang et al. [10] investigated H_2_O_2_, O_3_, and OH^●^ generation during electrolysis process. In this study, 21.2 mM H_2_O_2_ was generated, under 30 mA/cm^2^ of current density after 30 min of electrolysis. In another study by Wang at el.[34], in 60 min and 500 mA applied current, 800 mg/l H_2_O_2_ was generated.

### Survey of synergist effect of ozonation, electrolysis and photolysis

**Fig. 5:**
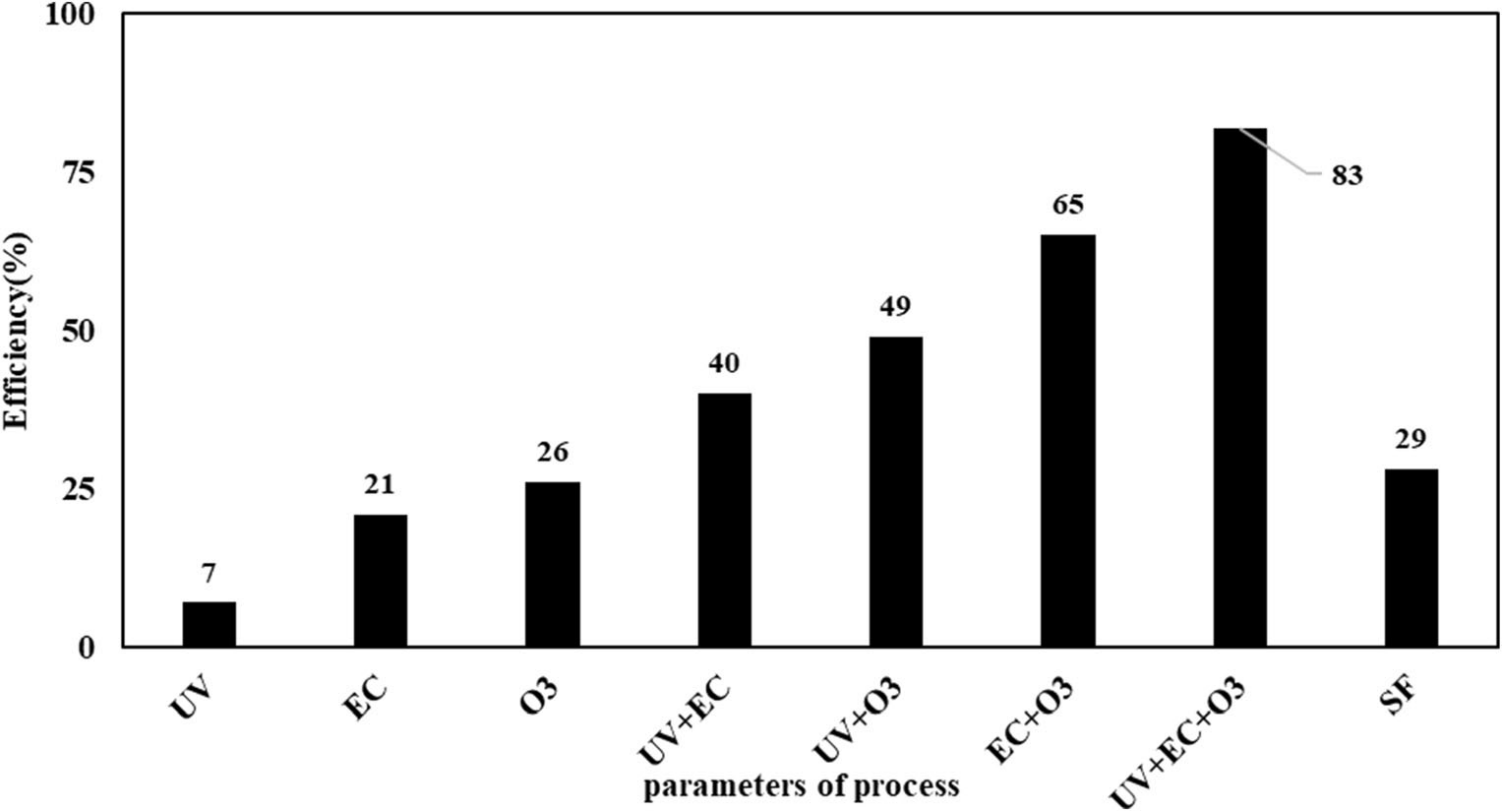
synergist effect of parameters on UV-EPP

Investigating the effect of every mechanism that involved in the UV-EPP process can well determine the relationship between the mechanisms. In this study, the main mechanism affecting on the UV-EPP are considered to be (1) the ozonation process in which ozone acts as a direct oxidizing agent and the radical hydroxyl activation factor, (2) the electrolysis process as anodic oxidation leading to production of hydrogen peroxide and radical hydroxyl and (3) photolysis. The results showed that each of the mechanism separately has a lower efficiency than their combination (Fig. 5).

According to experimental results, photolysis, electrolysis and ozonation process separately has 7, 21 and 26 percent of COD removal, respectively. The study by Mizutani et al. [19] found that the efficacy of the simple ozonation process and the electrolysis process separately in the removal of 1–4 dioxin as an indicator of organic compounds is much lower than when two processes are merged together.

### Energy consumption in UV-EPP

SER for COD removal in photolysis, electrolysis, ozonation and UV-EPP are presented in Table 6.

**Table 6:** Energy consumption in UV-EPP

	Process and mechanism	Condition	Energy requirment(kWh)
1	Photolysis	*Time = 100 min* *C0 and Ct = 9400 and 8742 mg/l respectively*	**0.09**
2	Electrolysis	*Time = 100 min* *C0 and Ct = 9400 and 7426 mg/l respectively* *A = 1 A,V = 10 V*	**0.5**
3	Ozonation	*Time = 100 min* *C0 and Ct = 9400 and 6956 mg/l respectively* *r = 10 kWh/kg o3 = 10* *ozone concentration = 29.11 mg/l.min*	**0.49**
4	UV-EPP	*Time = 100 min* *C0 and Ct = 9400 and 1692 mg/l respectively* *A = 1 A, V = 10 V* *r = 10 kWh/kg o3 = 10* *ozone concentration = 29.11 mg/l.min*	**1.5**

The report by Ahmadi et al. [31] investigated the specific consumption of energy in electrolysis, ozonation and E-peroxone process. In this research, as a merged procedure, the E-peroxone method not only considerably improved TOC removal but also significantly enhanced the energy efficiency for TOC elimination in comparison with the two separate procedures. In another report by Shen et al. [35] the energy efficiency was investigated. In this study, after 45 min of treatment, the UV/O_3_, EP, and PEP procedures eliminated 70%, 37%, and 98% of TOC from 1,4-dioxane solutions with SEC of 0.38 kWh/g TOC removed, 0.22 kWh/g TOC removed, and 0.30 kWh/g TOC removed, respectively

### Kinetic investigation

Kinetic investigation demonstrated that COD removal follows pseudo-first-order kinetics in UV-EPP. Particularly, the obvious COD removal rate constants (kUV-EPP) in the UV-EPP is meaningfully greater than the linear addition of the separate rates of resultant ozonation, photolysis, and electrolysis procedures. Kinetics can be reordered easily to an apparent first order equation (Eq. 17):

17 $$\text{ln}\left(\frac{{C}_{0}}{C}\right)=kt$$

**Fig. 6:**
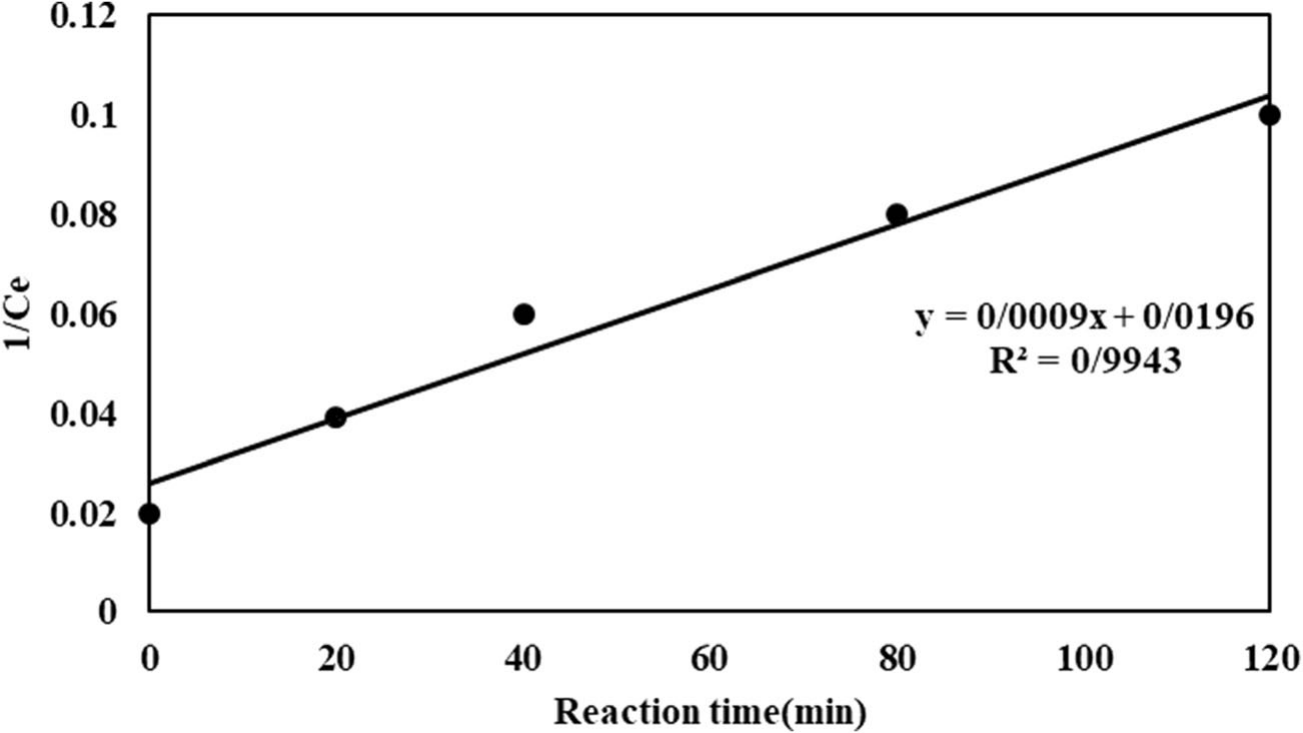
The first order kinetics of COD removal by UV-EPP

Where k_UV−EPP_ shows the first order rate constant of the COD removal. A plot of ln (C_ο_/C) versus time shows linear behavior, the slope of which upon linear regression equals the k_UV−EPP_. Usually, first-order kinetics are suitable for different investigations which practically well close-fitting by this kinetic model. Figure 6 represents the first order kinetics elimination of COD.

As demonstrated in Fig. 6, the removal of COD by UV-EPP was significant. Effectively, COD elimination due to the production of OH^●^.Also, Pearson coefficient and chi-square (Ҳ^2^) value were calculated and their values were set to 20 and 0.02, respectively. A report by Dominguez et al. [36] investigated the lindane degradation by the electro-oxidation process. In this research, the kinetics of degradation followed by a pseudo-first order reaction for lindane oxidation.

## Conclusion

Here in, we investigated the performance of COD removal via UV-EPP as a hybrid advance oxidation process. The results demonstrated that electrochemical activation of H_2_O_2_ significantly enhanced the removal of COD. The experimental data also confirm that UV-EPP as a hybrid advance oxidation procedure has a developed effectiveness than ozonation, electrolysis and photolysis, separately. In this case, the electrochemically and photocatalytic activated H_2_O_2_ increased the formation of OH^●^. Table 7 represent the summery of other reports on leachate treatment.

**Table 7:** Summary of the process for leachate treatment

Title	Operating conditions	Results and comments	References and Authors
Heterogeneous Fenton & electro-Fenton procedures	Iron-manganese binary oxide loaded zeolite (IMZ) was applied as a catalyst for producing OH^●^ in the solution.	88.6% COD from landfill leachate at the optimum situations. After Fenton treatment, biodegradability of landfill leachate was enhanced considerably from 0.03 to 0.52	[37]
Electrochemical/peroxydisulfate/Fe^3+^ treatment & ultrafiltration	The reactions were done in an electrolytic vessel with separated anode and cathode chambers and has been divided through a protonexchange membrane.	Anode/PS/Fe^3+^ Cathode/PS/Fe^3+^ procedure has the greatest impact on the organics destruction.	[38]
Integrated Electro-Oxidation/Electro-Coagulation/Electro-Reduction procedure	The influence of Factors such as leachate characteristics and procedure conditions on the efficiency of EO/EC/ER procedure was studied	simultaneous removal of carbonaceous and nitrogenous pollutants was attained in optimum conditions	[39]
Ozonation & supercritical water oxidation procedures	Ozonation was done at different reaction times (30–120 min). ScWO was studied at 600 °C, 23 MPa, and spatial time (τ) from 29 to 52 s.	A mixture of ozonation (30 min) and supercritical water oxidation procedure (O_3_-30’/ScWO) was the best method for the leachate degradation. These conditions allowed the great value removal of apparent and true color (92% and 97%, respectively), biochemical oxygen demand (BOD5,20) (95%), chemical oxygen demand (COD) (92%), total organic carbon (TOC) (79%), nitrite (78%), nitrate (84%), total (96%), dissolved (96%) and suspended (94%) solids.	[40]
Photo-Electroproxone Optimization and modeling in Leachtae Treatment	pH(4–8), DC(56/25–75 mA/cm2 ), ozone concentration(20–40 mg/l.min), Time(50–100 min),designed via CCD	pH = 5.6, ozone concentration = 29/1 mg/l.min, direct current = 74/7 mA/cm^2^, and reaction time = 98/63 min was determined. According to predicate point, the percentage of removal in optimum condition was 84/83 and 83 as a theatrical and experimental COD removal percentage via UV-EPP.	This study

- The optimum condition for UV-EPP is pH = 5/62, DC = 74/71 mA/cm^2^, ozone gas concentration = 29.11 mg/l.min and reaction time = 98/63 min. 83 percent of initial leachate COD was removed via UV-EPP in this condition.
- The reaction followed first-order kinetics model.
- The ozonation, electrolysis, and photolysis mechanism have lower efficiency in COD removal, separately. UV-EPP as a hybrid advance oxidation process have a synergist effect and increase COD removal in leachate.
- 29 percent synergist effect was obtained through applying hybridization.
- In the optimum condition of UV-EPP, 55/33 mM H_2_O_2_ was generated in an electrochemical process.
- The optimum 1.5 kW/h.kg COD electrical energy was required for leachate treatment.
